# Journalistic Denial of Death during the Very First Traumatic Period of the Italian COVID-19 Pandemic

**DOI:** 10.3390/bs11030041

**Published:** 2021-03-23

**Authors:** Sheldon Solomon, Daniele Rostellato, Ines Testoni, Fiorella Calabrese, Guido Biasco

**Affiliations:** 1 Psychology Department, Skidmore College, Saratoga Springs, NY 12866, USA; ssolomon@skidmore.edu; 2 Department of Philosophy, Sociology, Pedagogy and Applied psychology (FISPPA), University of Padova, 35131 Padova, Italy; daniele.rostellato@gmail.com; 3 Department of Cardiac, Thoracic, Vascular Science (DCTV), University of Padova, 35128 Padova, Italy; fiorella.calabrese@unipd.it; 4 Department of Specialistic, Diagnostic and Experimental Medicine (DIMES), University of Bologna, 40126 Bologna, Italy; guido.biasco@unibo.it

**Keywords:** COVID-19 outbreak, pandemic, terror management theory, moral panic, Corriere della Sera, denial of death

## Abstract

Exactly one year ago, between February and March 2020, the COVID-19 infection went from an epidemic confined to China to a worldwide pandemic that was particularly lethal in Italy. This study examined the media accounts during that period by analysing the representation of death-related constructs in Corriere della Sera, the most widely read newspaper in Italy. A textual and thematic analysis of articles published between period A (epidemic: 23 January–22 February 2020) and period B (pandemic: 23 February–31 March 2020) was conducted using Nvivo-11. A total of 141 articles comprising 48,524 words were collected. The most utilised words and meanings linked to COVID-19 were computed. In the rank distribution, ‘China’ and ’virus’ were the terms most frequently used in both periods. The terms ‘death’ and ‘dead’ were completely absent in period A and appeared in the 535th position in period B. The term ‘dead’ was used primarily to indicate the number of deceased. From a Terror Management Theory perspective, it is possible that the minimal reference to death-related issues was a reflection of death denial and a manifestation of efforts to deny death to manage terror. These findings highlight the ambiguities and ambivalence surrounding any issue pertaining to death; on the one side, undue alarmism may provoke exaggerated reactions, such as moral panic, while on the other denial-based messages that minimise references to mortality may reduce safe behaviour during a pandemic.

## 1. Introduction

Recent literature has emphasised how the global health system has developed sophisticated strategies to fight against infectious disease risks through formal and informal networks of organisations operating across the public, private for-profit and private not-for-profit sectors. Despite this, all countries continue to be attacked by infectious disease threats and potentially pandemics [1,2]. The capability of the global health system to afford effective protection against infectious disease threats was already called into question by the outbreaks of infections such as dengue fever, the Ebola virus, the Zika virus and Middle East respiratory syndrome [1,2] when in 2020 the world went through a significant crisis due to the COVID-19 (2019-nCoV) pandemic. The first cases were reported on 31 December 2019 when the Municipal Health Commission of Wuhan, China informed the World Health Organisation (WHO) that pneumonia of an unknown aetiology was occurring in the city. COVID-19 spread rapidly, threatening the health of large numbers of people, and thus would have required urgent action to stop the disease at the international level.

Before COVID-19, influenza pandemics had already caused significant mortal disruption for centuries all over the world. In the twentieth century, the A/H1N1 flu, the so called ‘Spanish flu’, pandemic was catastrophic. Scholars still debate the timing and the number of deaths that occurred. With a probable duration of two years and three waves, it caused approximately 40–50 million deaths [3]. Owing to the flu vaccination campaign in the past few decades, Western countries seemed to be able to prevent the problem of other disastrous pandemics. However, running in parallel with these strategies, globalisation and changes in human demographics have driven social transformations that have boosted the risk of the spread of viruses. In a pandemic outbreak of infection, without vaccines and safe therapies only behavioural strategies can prevent the infection from spreading. In this case, journalistic information might play an important role [4,5] in influencing people’s behaviour [6,7,8]. Scholars have shown that information on health and specific threats have an impact on human groups’ capability to prevent diseases. Indeed, insufficient or inadequate communication may hamper the strength of a society’s response to a public health emergency [9]. From the public health perspective, when a population is faced with a pandemic with possible high mortality, it is important to improve mass media activities because they might make the difference. Indeed, media can promote public awareness and increase public knowledge in order to cue the public to adopt protective behaviours [10]. The mass media acts as social amplification stations with respect to the perception of risk [11], and efficacious communication can help people adopt appropriate behaviours to stop the spread of the infection [12]. For example, some studies have shown how the fear aroused by exposure to information about MERS and influenza A H1N1 was positively correlated with increased risk perception and self-relevant behaviour [13,14,15]. With respect to a disease more similar to COVID-19, SARS, which appeared in 2002 with a lethality rate of 9.6%, was successfully contained by the summer of 2003 owing to the prevention information mainly managed by the media [16].

Emotional communication can only be useful if it is supported by clear and reliable information about the characteristics of the risk [17], and the media has a responsibility in this regard. However, infectious disease communication may encounter many difficulties, including uncertainty about the exact dimension of contamination in an outbreak’s initial stage and a lack of accurate information about the risk [14]. Moreover, COVID-19 is still unknown, because it is continuously evolving, then it would be important to correctly describe the aspects related to the risk of infection, such as dissemination, mortality, symptomatology. When this type of information is not clear and thoughtful enough, media communication can lead to an accentuation of anxiety and fear among the citizens [18]; this is another difficulty the media needs to manage.

Health communication during a public emergency creates some peculiar effects that are not always positive. In fact, the media has become an important factor in the dissemination of moral resentment and disdain, even if they are not expressly engaged in sensationalism [19]. With respect to health issues, the most important effect of this phenomenon is moral panic [20], which is a feeling of fear arising from social concern over some evil threatening people’s well-being. Moral panic occurs when a condition, episode, person or group of persons becomes defined as a threat to societal values and interests [20]. The phenomenon is characterised by increased anxiety about certain behaviours and sometimes stigmatisation of those who are considered responsible, despite the disproportionality of the actual risk [21]. Some moral panic phenomena, also called ‘folk devils’ (people or human groups who are blamed for various social problems) [20], have accompanied recent worldwide pandemics, including HIV/AIDS. Regarding HIV/AIDS, moral panic was engendered by designating this pathology the ‘gay plague’, a label that exposed heterosexual people to the risk of considering themselves immune due to their sexual orientation. Furthermore, the stigma resulted in discrimination against homosexuals and individuals with HIV/AIDS, including coercive and punitive measures such as quarantine, mandatory testing and the ‘branding’ of infected individuals [22]. On the one hand, moral panic is created by news media, which intensifies audience focus on a topic viewed as threatening through the use of stereotypes [23]. On the other hand, the news media may also underestimate a problem. This was the case during the Spanish flu (1918–1923), generated by a virus of subtype A/H1N1, which is considered the first pandemic influenza to have affected a ‘mass society’ [24]. In an attempt to avoid a lockdown, educational strategies were adopted utilising newspapers as the main vehicle of information. Nonetheless, the mandate at that time was to scale down collective concerns with the slogan ‘Don’t panic!’ [24]. If it is true that the overestimation of risk can lead to severe social and economic consequences, the effect of underestimation can be equally devastating. According to recent studies, the number of estimated deaths worldwide due to the Spanish flu is 20–50 million [25]; this is why influenza viruses are now considered among the main health threats today [26]. However, it is also important to note that this pandemic affected the whole world, but it is still called the ‘Spanish flu’. This national label is due to the fact that the only newspapers that informed the population were the Spanish ones. On the contrary, the media of other countries preferred to deny the seriousness of the local situation, attributing the problem to Spain only [24,25,26]. 

The importance of the traditional press and professional journalism is that readers expect reliable information from them. Their role in recent years has been further enhanced due to the explosion of unreliable news circulating on the Internet that is an increasing source of health information worldwide. A plethora of studies have shown how use of the Internet is a risk to public health. In particular, COVID-19 triggered a viral dissemination of false or misleading information, and by 6 February 2020 no quality information on the topic was available on the Internet. Scholars therefore suggest that governments should regulate this form of health information [27]. An infodemic is an overabundance of accurate and/or inaccurate information occurring during an epidemic, spread via digital and physical information systems. The inaccuracy is related to false perceptions and fake news using emotional language and conspiratorial reasoning. An infodemic makes it very difficult for people to find trustworthy sources and reliable guidance when they need it [28]. As emphasised by The Lancet [29], immediately after COVID-19 was declared a Public Health Emergency of International Concern, the WHO activated a dedicated platform called WHO Information Network for Epidemics (EPI-WIN), the aim of which is to reduce the infodemic effect and share tailored information with specific target groups [30]. The use of clear and positive language, the necessity of anticipating and managing misinformation and engaging with media outlets were considered crucial [31]. 

The difficulties of media health communication are related to the fact that panic is the result of the fear of dying. Indeed, death is the most sensational factor that motivates people to protect themselves, and this is why it is important to study how the news considers this issue. In the area of communication theory aimed at explaining how the various contextual frameworks are constructed and transmitted, Terror Management Theory (TMT) gives a compelling account of why these frameworks are psychologically significant [32,33,34]. TMT states that the unique human awareness of death gives rise to potentially debilitating existential terror that is ‘managed’ by embracing cultural worldviews that confer a sense that one is a person of value in a world of meaning (i.e., self-esteem) and therefore eligible for literal and/or symbolic immortality. Two levels of defence are activated in response to intimations of mortality: (1) ‘proximal defences’ directed at keeping explicit awareness of mortality outside of focal awareness via denial, distraction or rational instrumental behaviour and (2) ‘distal defences’ to bolster faith in one’s cultural worldview, boost self-esteem and fortify relationships with significant others [35,36]. More than 1000 TMT studies demonstrate the pervasive influence of death reminders on people’s attitudes, feelings [37,38] and behaviour, including fostering in-group bias and amplifying prejudice and discrimination against out-groups [39,40]. Improving the accuracy and effectiveness of communication regarding existential issues is therefore particularly important because these issues are likely to evoke conscious and/or unconscious concerns about death and to stimulate proximal and distal terror management defences [41,42]. 

Italian communication strategies and risk awareness protocols are in line with the WHO [43], structured according to the transparency of information and the definition of appropriate communication strategies. On 19 March 2020, the newspaper Corriere della Sera (CdS) published a photo showing a long line of army trucks transporting piles of coffins from Bergamo (a city in northern Italy) to another region for cremation, using the term ‘death’ only once and ‘dead’ twice. On the same day, a video describing the scene went viral on the Internet. Two weeks later, the Ministry of Health (MdS, 2020) announced that suspected fatalities from the virus did not require post-mortem examinations. This was contrary to autopsy research, which could have provided a crucial understanding of why the virus was so lethal. A discussion ensued among researchers to explain this apparent anomaly. One possible explanation from a TMT perspective was that the relatively scant allusion to death in the Italian media and the Health of Ministry’s disinclination to engage in post-mortem examinations of victims at the outset of the pandemic could be a reflection of proximal and distal terror management defences used to quickly foreclose the glaring and burgeoning evidence of death. The present study was therefore undertaken to understand if and how unconscious defensive strategies were in place at a linguistic level to reduce or eliminate the salience of widespread mortality during the transitional period when COVID-19 passed from being an epidemic to being a pandemic. Could effective and timely journalistic communication have prevented this transformation?

It is certainly impossible to know if a good piece of information that alarmed the Western population in the right way could have prevented the spread of the virus, particularly in the very early stages of the contagion from China to Italy and then to the rest of Europe and the world. However, in light of TMT studies, improving the effectiveness of communication could be significant in better managing the information for preventive purposes. Indeed, it is important to avoid adopting strategies to reduce risk that may paradoxically encourage the spread of the virus [44]. This article presents qualitative research aimed at analysing journalistic representations of the virus in relation to its associated mortality in Italy during the critical time when the COVID-19 epidemic became a pandemic. The fundamental hypothesis is that a moral panic effect was partially created in the first phase and then withdrawn in the second phase because of the need to deny mortality. As indicated by the studies on media influence and contemporary communication, TMT, which emphasises the biased ways individuals keep the fear of death at bay, is useful in understanding the results of this study and in improving the comprehension of contemporary communication on COVID-19. The clinical relevance of this research is due to the fact that good media communication allows people who face the danger of contagion in an emotionally balanced way to avoid behaviours that may harm their health. In fact, the study considers a topic that has not yet been sufficiently addressed by the international literature studying the relationship between media communication and emotion management in the face of pandemic risk. In particular, the theme of the fear of death and how the removal of topics concerning it can produce social risks that must be taken into account are analysed in an innovative way. With respect to clinical and therapeutic work, this research highlights how some attitudes of denial and removal of death are not only individual but characterise the whole society. Detecting this can help the clinical work allowing to shed light on an aspect still very little studied.

## 2. Materials and Methods

The main aim of the study is to recognise if and how COVID-19 mortality was presented in the first months of the pandemic in the most important Italian newspaper. The second objective is to describe whether the mortality risk associated with the virus was sufficiently emphasised and how or if secondary aspects of the contagion were emphasised instead. The third objective is to ensure that the texts considered were the most representative of a well-considered and accurate information base to maximally reduce the risk of any infodemic effect characterised by inaccuracy, as was already evident since the first months of Internet coverage. The ultimate goal is to consider some useful aspects related to the communication of death to better manage the very first moments of future pandemics and thus highlight the necessity of training politicians and health system and communication professionals to better manage information about the risk of death when the first signs of a potentially pandemic infection appear.

The study adopted a qualitative research design, which is considered a reliable method of investigation, particularly in relation to issues for which the semantic contours have not yet been fully defined, as exists in a pandemic scenario. Specifically, the main objective of the qualitative analysis was to examine the ways in which the relationships between representations of COVID-19 and of death were managed. To this end, textual analysis was used, more specifically a subset of categories or representational images in CdS, the most popular newspaper in Italy and traditionally regarded as the most moderate in terms of information management. 

Despite the fact that COVID-19 was given pandemic status by WHO on 3 March 2020, the three stages of the virus propagation in Italy can be divided as follows: (1) First outbreak (January)—from the first cases in Wuhan to the spread of the virus in China and the appearance of isolated cases in other countries until WHO declared a state of ‘global health emergency’; (2) epidemic (February)—the new European outbreak and the first prevention plans as the virus spread rapidly in several countries, including Italy; and (3) pandemic (March–April)—the virus became a global phenomenon, new containment measures were implemented inspired by the ‘Chinese model’ and drug trials began. 

The study identified two periods of time that were useful in collecting the newspaper articles: period A (21 January–21 February) and period B (22 February–31 March). A total of 142 articles were collected, 110 of which originated in period B. Articles were selected based on the presence of certain keywords (e.g., ‘virus’, ‘emergency’, ‘health’, ‘China‘, ‘Hubei’, ‘death’ and ‘dead’) and on their position, favouring articles in which these words appeared higher on each page.

The exploratory procedure utilised NVivo-11, computer-assisted qualitative data analysis software (QDAS) that ensures various possibilities for interaction between the data and that is useful in qualitative research designs. The use of QDAS improves the process of learning from data and increases the efficiency in tracing the most important contents. The choice of Nvivo for this study was suggested by the fact that the amount of textual data was vast and potentially disorganised. Generally, QDAS helps manage data and allows an increased focus on ways of examining the meaning of what is being considered. In this study, it was particularly significant to highlight the removal of all the terms related to death, and Nvivo was particularly efficacious in that regard. Furthermore, it permits one to easily trace quotations and statements on the basis of keywords to better manage the thematic analysis focused on specific questions. The analysis was divided into five phases: (1) Lemmatisation of the texts, coding articles, recognition of the units of analysis inherent to the relationship between COVID-19 and death and selection for their relevance; (2) selection of all the quotations useful in identifying a range of examples of the theme, production of the matrix of frequencies illustrating the two periods and two word clouds representing the two periods in Italian; (3) generation of insights through the interpretation of the articles in their entirety and the personal point of view of the researchers to ensure the coherence of the overall presentation and its relevance to the data; (4) translation of the matrixes into English and artistic transformation of the two word clouds though Word Art into impact images able to represent the sense of the analyses; (5) final discussion of the issues by the researchers and revision of the manuscript in a manner that remained faithful to the content of the articles while enhancing the themes; and (6) a final meeting with all the researchers and with the CdS director.

## 3. Results

For period A (epidemic), the total number of articles was 32 and the total number of words was 17,804. For period B (pandemic), the total number of articles was 110 and the total number of words was 66,720. After lemmatisation, 955 significant words were selected. Table 1 shows the 50 most used words, their frequency and their percentage of the total number of words of each corpus for the two periods. The term ‘death’ is absent in period A and appears in period B 12 times in the 535th position.

In period A, the most prominent allusions were to ‘China’ (frequency *n* = 204), in particular ‘Wuhan’ (104) and ‘Beijing’ (74), with emphasis on the number of ‘cases’ (50) and ‘people‘ (41) involved (‘millions’ (27)) and how ‘cities’ (41) reacted to ‘quarantine’ (27). Comparisons were made between the present situation (‘now’: (31)) and the previous ‘SARS’ epidemic (32). Rome (26) appeared as an Italian city because its hospital admitted two sick Chinese ‘tourists’ (18). ‘Fear’ (22) assumed the same relevance as the ‘mask’ (21) necessary to protect health and ‘symptoms’ (19) of the disease. The word ‘death’ (0) was completely absent and ‘dead’ (18) was used solely to indicate the number of deceased. 

In period B, when the infection changed from an epidemic to a pandemic, the prevalent themes were centred on ‘policy’ (388) and ‘measures’ (107) adopted to fight the ‘virus’ (347) in the ‘region’ (138), particularly in ‘Northern Italy’ (283) because it had the most infections. Other themes included the commitment of the ‘government’ (110), the position of the ‘president’ of the republic (79) and the relationship of ‘Italy’ (101) with other countries (‘country’:176). The articles highlighted the ‘number’ (70) of ‘cases’ (243) of infection (‘infection’:128); ‘infected’:67), indicating in parallel the number of ‘deaths/dead’ (111) on the different ‘days’ (240). There was an emphasis on the ‘protection’ (78) of ‘health’ (65), the need for ‘medical devices’ (125) and the necessity for all people (‘people’: 109; ‘all’: 76; ‘everybody: 144) to stay ‘home’ (91) in ‘quarantine’ (65). The condition of ‘doctors’ (103), ‘hospitals’ (63) and ‘patients’ (80) was described, emphasising the present contingent moment: the ‘situation‘ (77); ‘now‘ (71); the ‘moment‘ (69). The word ‘risk’ was used 58 times and ‘death’ 11 times (Table 1).

### 3.1. The First Thematic Area: The Chinese Situation

**Figure 1:**
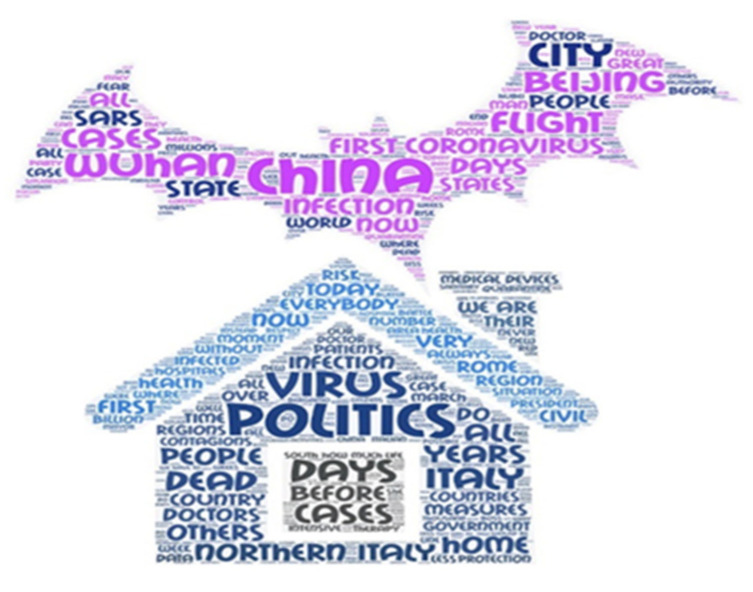
Word cloud, Periods A and B: ‘Stay home: politics saves you from the virus’.

The first theme characterised the first period. The term ‘dead’ was mainly used in the context of the confirmed number of dead and the percentage of increase and time. In the first period, all news referred to the situation in China: ‘Exceeded the number of sick SARS [...] and there are the first infected foreigners [...] The less bad news is that the many deaths are still a sixth of the great epidemic of SARS and Tuesday would have decreased the new daily contagions’ (CdS, 30 January); ‘Western epidemiologists estimate that “1500 deaths must be expected in the end”, twice as many as the SARS epidemic 2002–2003′ (CdS, 29 January). The reconstruction of the possible origin of the infection was linked to exotic animals in markets: ‘at the marketplace, fish, birds and animals, more or less exotic, slaughtered or caged. Controlled beef and pork, exhibited next to live and dead animals of uncertain origin. A big business [...] The “patient zero”, identified on 8 December in Wuhan, had been in that big market’ (CdS, 27 January). Indeed, the representation of the virus in this phase was related to bats and snakes: ‘Scientists hypothesise that the 2019nCoV virus arrived to humans from two species of snake, the bandaged bungaro and the Chinese cobra, which in turn had been infected with a similar disease by bats’ (CdS, 24 January); ‘It is very similar to the Bat Coronavirus, so it is likely that these animals transmitted it. Between them and humans, an intermediate host, most likely in the fish market’ (CdS, 29 January); ‘But badgers and mice, bats, civets, marmots, otters, wolf cubs are also suspected. The Chinese like ye wei, which can be freely translated as “wild taste”’ (CdS, 27 January); ‘It was discovered that the coronavirus of the time had passed from bats to civets, animals of the proportions of a cat. There is a half Mandarin proverb, in which one boasts of being able to eat “everything that has four legs, flies or swims” (“except tables, planes and boats”)’ (CdS, 27 January). Finally, in the counts of the dead, there also appeared representations of COVID-19 as a devil: ‘The epidemic is a demon, we won’t let a demon stay hidden’ (CdS, 29 January). Figure 1 shows the word cloud for period A with the image of a ‘Chinese diabolic bat’. 

### 3.2. The Second Thematic Area: Death as a Shocking Number but Limited to Some Categories

The infection was considered ‘a fact which, however, does not alarm the experts, because for the moment half of them are asymptomatic or with a simple cold [...] As far as the deaths are concerned, these are people of high age and who have a series of previous pathologies’ (CdS, 29 February); ‘As far as the deaths are concerned, these are people who are older and have a number of prior illnesses’ (CdS, 29 February). However, the sudden increase in deaths soon changed the scenario: ‘There are no hugs from friends or distant relatives, nor wreaths of flowers in front of the churchyard or notebooks to record pain and participation […] These 52 dead (a number destined perhaps to grow in the night) have disappeared from the accounts of the living without making a sound, in some cases without the comfort of a prayer’ (CdS, 3 March). Particular attention was paid to the prison issue: ‘There’d be a jailhouse emergency commissioner […] The minister Bonafede stated, “I do not think it is normal at a time like this to have 30 prisons in revolt with dead, wounded and escaped prisoners”’ (CdS, 11 March). This theme was especially developed in the narration of the demoralizing effect of defeat: ‘In the face of the realities of the infected and the dead, separate the essential from the superfluous. With one goal—to keep people indoors as long as possible—Lombardy is asking for the closure of shops, factories’ (CdS, 11 March); ‘Now there’s nobody on the streets of Bergamo. It’s discipline, but also fear. The province has the record for being infected by coronavirus (1472) [...] What is the scariest thing today?’; ‘The growth of contagion doesn’t stop. Hospitals should be thanked for the incredible effort they’re making’ (CdS, 11 March); ‘There it is, tomorrow. It’s in the shocking numbers of the infected and the dead, it’s in the very strong pressure of Lombardy at the end of its life, in the terror of the governors of the South, in the thousands of messages via social media by imploring citizens’ (CdS, 12 March); ‘The people dying in clusters, that was a failure. Too many dead […] We wanted to defend Toyland and the economy even in the face of death’ (CdS, 25 March); ‘Italy has more dead than China. We reached the sad record yesterday with 427 more deaths in 24 h’ (CdS, 20 March). Furthermore, the “defeat” was represented as the phase after war: ‘When all will be over— the entrepreneurs say—it will be after the war’ (CdS, 31 March).

### 3.3. Third Thematic Area: The Virus as a Political Issue

With respect to the Italian situation, which by the end of February was dire, political issues prevailed in the news reports: ‘a clumsy ordinance (“We would risk 1200 deaths”) [...] Italy at the time of the coronavirus continues to move in scattered order’ (CdS, 27 February). Political topics spanned every field, from economics to culture, from parliamentary conflicts to mediation and from international to local issues; ‘there are no more visits to the plant and now transport is also being blocked’, says Stefano Scaglia, CEO of the group of industrial automation systems’ (CdS, 2 March); ‘The government prepares a decree with a package of measures for 3.6 billion to boost the economy’ (CdS, 2 March); ‘But then, we want a plan of structural growth for the country, for which strong political cohesion is needed’ (CdS, 2 March); ‘In essence, the alarms launched by the French President Emmanuel Macron, the German Minister of Health, Jens Spahn and the Minister of the Economy, Roberto Gualtieri of the Democratic Party, who asked the EU for more budgetary flexibility for Italy’ (CdS, 3 March) (Figure 1). 

In response to the results of our study, the director of the CdS stated: ‘There is no planned strategy or rule that requires journalists not to use words related to death’; ‘There has always been an empathic sensitivity to readers on the part of the entire editorial staff, aimed at reducing anxiety’; ‘After March, CdS began to give names and describe biographies to remember the dead and not just count them’. Furthermore, he emphasised that journalists tried to empathise with their readers by presenting the contagion and its mortal risk in a moderate way. The main aim was to control any panic effect, preventing possible adverse consequences and balancing the emotional dimension with the exigence of truth. Regarding the moral panic, he observed that in reality the word China did not refer so much to the Chinese as a cause but to China as a necessary containment area. The journal wanted to indicate from the start the necessity of confining the contagion.

## 4. Discussion

As highlighted by Eysenbach [28], the infodemic effect that characterises Internet makes it very difficult for people to find reliable information that can guide their health behaviour. Traditional media and journalism try to explain information in a moderate way, to offer more reliable indications, particularly with respect to health. The international literature has extensively highlighted the problem of the infodemic in managing information related to COVID-19 since the very first months of the pandemic. Studies on the ways in which the media have handled the COVID-19 pandemic have already accounted for some important aspects, including the biases that characterise mass information [45] (AlAfnan, 2020), among which stigma and incitement to hatred emerge in importance [46] (Robie, Krishnamurthi, 2020), but have also underlined the ability to give scientifically correct information [47] (Anderson et al., 2021). They then considered effects from the resulting increase in perceived risk of contagion as well, including depression [48] (Olagoke et al., 2020), worry and fear [49,50] (Garfin et al., 2020; Manzoor & Safdar, 2020). Despite all these contributions, to date there is very little literature regarding how the press has addressed the current pandemic. Our study considers a topic that has never been addressed in either COVID-19 or previous pandemic-related analyses. In particular it wanted to investigate how the denial of the mortality of the virus acted in the most important Italian newspaper.

Indeed, there are not yet specific studies that consider the ways in which the death message is handled in pandemic cases, particularly during the first months of contagion. The importance of this topic is underscored by the fact that the subject of death has a strong emotional impact and can create panic. If not considered thoughtfully, there is a risk of underestimating an important public health concern. 

This is the first study to consider how death is treated by the mainstream press in the early months of a pandemic and to reflect on why it is important to develop an efficacious strategy to manage this issue when an infection endangers public health. The qualitative research was focused on the problem inherent to the way the most important and widely read Italian daily newspaper (CdS) managed information in the first two months of the COVID-19 outbreak. This period was chosen because it could be crucial in managing the next phases of the problem, both in terms of the contagion itself and the organisation of social life. Three main results emerged from the quantitative analyses of the data: in period A, when the virus did not directly affect Italy, the word ‘death’ was completely absent and moral panic took an embryonic form, without demonisation, whimsically attributing the epidemic to a Chinese preference for eating the meat of wild animals. 

The transition from depicting the virus as resulting from Chinese ‘wild taste’ to the representation of a radical ‘otherness’ demarcated a psychological boundary and served as a precursor of moral panic; both stages were linked to cultural differences. At the outset of the epidemic, viewing the virus in terms of the strange tastes of ‘primitive’ people with an alien culture (‘them’) was a potent defence against death anxiety; Italians with modern tastes linked to ‘our’ local cultural tradition need not worry about becoming infected. However, when the virus became prevalent in Italy (and Europe and the US) and the death toll rose precipitously, Asians in general and Chinese in particular became targets of racial and xenophobic animosity [51]. Similarly to what happened with the Spanish flu, Covid-19 has been nicknamed the ‘China virus’. The process of labelling reduces the willingness to engage in empathetic behaviour towards those who are suffering. As social psychology demonstrates, the process of labelling is functional to victim blaming and this saves the effort of responding to victim’s need for help [52]. Indeed, the infodemic term ‘China virus’ was propagated across the Internet, conveying different expressions of racism [53]. This difficulty culminated on 29 May 2020 when President Donald Trump announced the US would leave WHO [53], holding China solely responsible for the entire pandemic, even though the British journal Nature [54] had apologised in April for falsely relating the virus to China and Wuhan. In this way, the journal wanted to stand against the stigmatisation of Chinese people because of COVID-19 and any actions that associated viruses with specific locations. The journal called for a stop to the racism and discrimination against people from South East Asia that had emerged in the early days, thus opening a discussion on the stigma in the scientific community, whereas scholars begun to consider similarities between disease stigma surrounding infectious diseases, such as HIV, TB or leprosy and COVID-19 [55,56,57,58,59]. However, after Nature’s declaration, CdS no longer made explicit reference to cultural aspects that characterised Chinese cuisine. As already underlined by the literature [49,55,56], these early signs of stigma attached to COVID-19 somehow intersected with the existing prejudices related to migratory processes. Worldwide, misinformation on the Internet fed by conspiracy theories was particularly prevalent with respect to COVID-19 and supported the construction of stigma as a defensive strategy, as widely demonstrated by TMT in similar situations where mortality salience was particularly intense [32,33,34,35,36,42]. Because these psychosocial dynamics can reduce healthcare engagement and adherence to public health practices, as already demonstrated with HIV, and because they transform concrete action aimed at protecting public health into blame against those who are perceived as guilty [49,55,56], it would be particularly significant in the future, when other pandemics will plague the world, to find a communication strategy useful to prevent the stigma effect in the early stages of contagion.

In period B, when Italy was overwhelmed by the pandemic, the word ‘death’ emerged but in a somewhat muted fashion; the word ‘dead’ appeared more often, albeit relatively infrequently and almost entirely to indicate numbers. Conversely, political discourse became preponderant.

These findings are potentially reflective of a biphasic manifestation of death denial in the media. In period A, during the epidemic, CdS reduced moral panic by eliminating reference to the word ‘death’. Simultaneously, by attributing the virus to ‘Chinese wild taste’, Italians could reduce existential distress by perceiving themselves as immune to the virus by virtue of geographical distance and culinary preferences, that is ‘cultural superiority’. These are, from a TMT perspective, proximal defences to remove death thoughts from explicit awareness.

In the second phase (i.e., the pandemic), moral panic dissipated as media coverage of COVID-19 was framed primarily in political terms. The word ‘death’ was submerged under the preponderance of discourse by politicians and institutional figures who dominated the daily news consumed by Italians. This could serve both as a proximal defence to ward off death anxiety by focusing citizens’ attention on ‘business as usual’ and as a distal defence by bolstering confidence in Italy’s political, economic, medical and cultural institutions. 

Consistent with this terror management account of journalistic death denial, the CdS director confirmed that the newspaper was explicitly devoted to reporting about COVID-19 in a manner that minimised readers’ anxiety and stress. In a long discussion about the results of this research and to understand the direction the newspaper wanted to follow, it became clear that the fundamental principle was to keep ‘the great boat of Italy’ afloat and to avoid allowing general panic to sink it. Because CdS is the most widely circulated and highly regarded newspaper in Italy, this was likely the best strategy to limit the psychological damage caused by the pandemic. However, it is important to remember that during the first pandemic influenza affecting ‘mass society’, the Spanish flu (1918–1923), the slogan ‘Don’t panic!’ may have reduced anxiety levels among the population. However, even though the media provided information about the importance of taking measures to limit contact [24], the global death toll of 20–50 million was devastating.

## 5. Conclusions

It is difficult to stop a pandemic when it explodes, and certainly the best thing is to recognise the signs of danger as soon as they begin to appear. In this process, the dynamics of social information in the early stages are crucial, and it is important to recognise the threat signals immediately. The globalised world is based primarily on information and the facilitation of forms of contact and communication. From this, two important factors emerge for the promotion of public health—the possibility of early knowledge of the risk of a contagion outbreak in order to curb it and the possibility that the news that is disseminated may be misleading and therefore not useful in stopping the contagion. Analysing the forms of media communication in the early days of a pandemic can help us to understand what processes were put in place and to understand how they worked. For this reason, it is important to study how the news is made available to citizens by the media in the very early stages of a pandemic. Our analysis of the articles in Italy’s most important national newspaper shows that in this regard the problem for journalism is how to manage existential terror effectively while also conveying accurate information about mortality risks and how to prepare people for these kinds of experiences, which may become increasingly frequent because of globalisation. This means that conveying widespread information on death and dying could be essential.

The issue of information management related to the risk of death requires further study to corroborate these findings and to delineate how various forms of death denial affect journalistic expression. On one hand, there is the risk that alarmism activates dysfunctional defensive reactions, as happened with HIV/AIDS (moral panic and discrimination against minority groups). On the other hand, dampening intimations of mortality to diminish anxiety levels results in providing inadequate warning to the population. At present, finding the balance is entrusted to the empathic capacity of journalists. It is very important to include specific reflections on fear of death and psychological defensive strategies in communication science training courses [41]. Particular attention should be paid to cultural thanatology—that is, studies related to the cultural ways in which death-related issues are handled—in the field of health work and in the spaces of policy construction. The total absence of reflection on the mortality associated with the virus by CdS in the very first months of the contagion showed how, especially in the political dialogue, the problem was not considered and focused on at all. The importance given to the discussion of politicians highlighted how much they did not consider at all the risk of death that the contagion implied. This result highlights the need for politicians to become aware of the psychological dynamics that lead to the removal of death from conscious thought. Knowing how to recognise a mortal risk to public health in time is perhaps the first task of a government. Moreover, the same kind of training should be provided for health professionals, who are the first to pick up signals of potential danger. Indeed, they are the ones who have the basic training to recognise the presence of a potentially deadly new phenomenon. They in turn should be trained to develop this sensitivity to the best of their ability and to organise themselves in such a way as to be able to manage the message about the risk of death to political bodies in a coordinated manner. Finally, appropriate consultation between political bodies, health authorities and the media on these issues should prompt new policies and international guidelines for recognising the danger and alerting the population in time and speaking about death appropriately, promptly and without reticence. 

The main limitation of the study is that that data were drawn from only once source, CdS, for the first two months of the pandemic. Future research could consider other important newspapers and comparing the news published by the traditional press with the information on the most popular Internet sites for a period of time that includes subsequent phases of the pandemic. In addition, future research could consider the relationship between information circulating on the Internet and the traditional press. In fact, the traditional press is considered a space for reworking what is presented on the social media. This would allow emphasising the latent processes concerning in particular the unconscious defensive dynamics for the reduction of anxiety caused by mortality salience, such as the process of stigma construction. Future studies could consider the similarities and differences in the management of death communication in different countries. A further limitation of the research is that it did not systematically explore the viewpoints of the CdS director and of the managing editors to compare their intentions with the effects of the information produced by the newspaper. Future studies could detect any similarities or discordances, and this could be useful for the improvement of the quality of the media communication.

## Figures and Tables

**Table 1:** Frequencies of the first 25 words in the two periods.

Period A				Period B		
Word	Frequency	%	*n*	Word	Frequency	%
China	204	1.146	1	Politics	388	0.586
coronavirus	162	0.91	2	virus	347	0.524
Wuhan	104	0.584	3	Northern Italy	283	0.428
Beijing	74	0.416	4	cases	243	0.367
infection	71	0.399	5	days	240	0.363
flight	53	0.298	6	infection	214	0.323
cases	50	0.281	7	Country	176	0.266
city	41	0.23	8	doctor	161	0.243
people	41	0.23	9	everybody	144	0.218
health	41	0.23	10	region	138	0.208
days	37	0.208	11	before	137	0.207
state	37	0.208	12	medical devices	125	0.189
States	36	0.202	13	Hospital	114	0.172
first	34	0.191	14	dead	113	0.171
sars	32	0.18	15	government	110	0.166
Now	31	0.174	16	people	109	0.165
doctor	31	0.174	17	measures	107	0.162
world	29	0.163	18	others	102	0.154
all	29	0.163	19	Italy	101	0.153
great	28	0.157	20	years	91	0.137
millions	27	0.152	21	home	91	0.137
before	27	0.152	22	today	83	0.125
quarantine	27	0.152	23	patients	80	0.121
Rome	26	0.146	24	president	79	0.119
situation	26	0.146	25	protection	78	0.118
man	26	0.146	26	situation	77	0.116
New Year	24	0.135	27	Rome	75	0.113
where	23	0.129	28	very	72	0.109
authority	22	0.124	29	Now	71	0.107
fear	22	0.124	30	number	70	0.106
case	21	0.118	31	moment	69	0.104
control	21	0.118	32	without	69	0.104
new	21	0.118	33	infected	67	0.101
mask	21	0.118	34	health	65	0.098
home	20	0.112	35	quarantine	65	0.098
people	20	0.112	36	first	64	0.097
Italian	20	0.112	37	civil	62	0.094
party	20	0.112	38	they	62	0.094
all	20	0.112	39	March	61	0.092
others	19	0.107	40	over	59	0.089
Hubei	19	0.107	41	risk	58	0.088
always	19	0.107	42	billion	56	0.085
symptoms	19	0.107	43	time	56	0.085
dead	18	0.101	44	health	55	0.083
tourists	18	0.101	45	case	54	0.082
minister	17	0.095	46	intensive	53	0.08
moment	17	0.095	47	week	52	0.079
today	17	0.095	48	therapy	51	0.077
weeks	17	0.095	49	battle	51	0.077
…	-	-	-	…	-	-
death	0	0	535	death	11	0.017

## Data Availability

Not applicable.
